# Structure activity relationship and modeling studies of inhibitors of lysine specific demethylase 1

**DOI:** 10.1371/journal.pone.0170301

**Published:** 2017-02-03

**Authors:** Chao Zhou, Fangrui Wu, Lianghao Lu, Liping Wei, Eric Pai, Yuan Yao, Yongcheng Song

**Affiliations:** Department of Pharmacology, Baylor College of Medicine, 1 Baylor Plaza, Houston, TX, United States of America; University of East Anglia, UNITED KINGDOM

## Abstract

Post-translational modifications of histone play important roles in gene transcription. Aberrant methylation of histone lysine sidechains have been often found in cancer. Lysine specific demethylase 1 (LSD1), which can demethylate histone H3 lysine 4 (H3K4) and other proteins, has recently been found to be a drug target for acute myeloid leukemia. To understand structure activity/selectivity relationships of LSD1 inhibitors, several series of cyclopropylamine and related compounds were synthesized and tested for their activities against LSD1 and related monoamine oxidase (MAO) A and B. Several cyclopropylamine containing compounds were found to be highly potent and selective inhibitors of LSD1. A novel series cyclopropylimine compounds also exhibited strong inhibitory activity against LSD1. Structure activity relationships (SAR) of these compounds are discussed. Docking studies were performed to provide possible binding models of a representative compound in LSD1 and MAO-A. Moreover, these modeling studies can rationalize the observed SARs and selectivity.

## Introduction

Gene transcription is regulated by post translational modifications of histone proteins, which mostly include methylation and acetylation of a lysine or arginine sidechain.[1] The resulting histone steric and/or electrostatic alterations lead to the formation of a transcription protein complex that directly controls gene expression. Recently, aberrant histone modifications are frequently observed in many types of cancer and histone modifying enzymes are therefore considered potential drug targets.[2–4] Lysine specific demethylation 1 (LSD1) can remove the methyl group from a mono- or di-methylated lysine residue of histone H3 lysine 4 (H3K4), H3K9 or a non-histone protein.[5–7] The biological function of LSD1 is crucial, as LSD1 knockout in mice was found to be embryonic lethal, while conditional knockout blocked hematopoiesis.[8] Overexpression of LSD1 was found in a broad range of cancers, including lung, prostate and breast cancers.[9–11] Recently, LSD1 has been reported to be a drug target for acute myeloid leukemia (AML).[12–14] AML is the major type of acute leukemia, showing a poor prognosis with 5-year survival rates being only 24.6%.[15] Current treatments are mostly conventional chemotherapeutics, which non-selectively kill all rapidly dividing cells including normal cells in bone marrow and other organs. This causes severe toxicities and side effects that significantly limit the efficacy of these drugs. There is therefore a pressing need for new therapeutics to treat AML.

**Fig 1:**
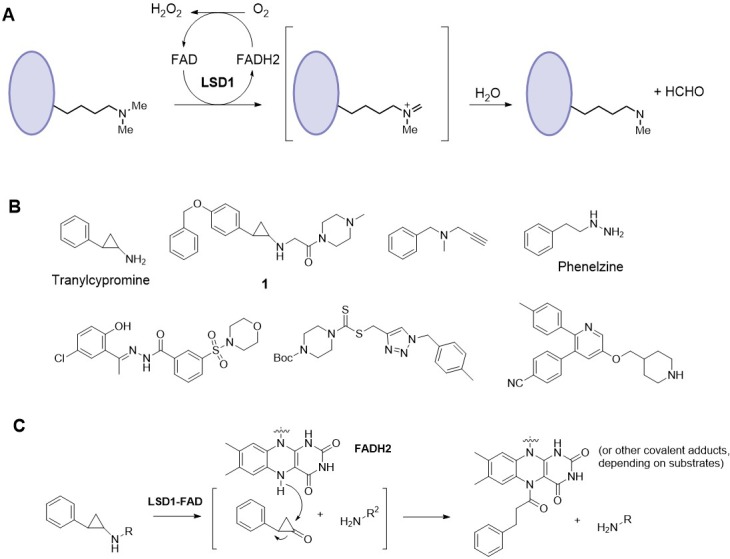
(**A**) Mechanism of catalysis for LSD1; (**B**) Structures of representative LSD1 inhibitors; (**C**) Mechanism of cyclopropylamine containing LSD1 inhibitors.

LSD1 belongs to a family of flavin adenine dinucleotide (FAD) dependent monoamine oxidases (MAO), with its mechanism of catalysis shown in Fig 1A.[16] FAD oxidizes the methyl group of a substrate, e.g., H3K4-Me1 or 2, to generate an imine intermediate, which is hydrolyzed to produce the demethylated product and formaldehyde. The reduced form of FAD is oxidized by O_2_ in the solvent to complete a catalytic cycle. A number of LSD1 inhibitors with several chemotypes, including cyclopropylamine, propargylamine, hydrazine, triazole-dithiocarbamate and 3,5,6-substituted pyridine, have been reported in journals and patents,[17–26] as representatively shown in Fig 1B. The majority of the current LSD1 inhibitors contains a cyclopropylamine core structure, which upon oxidation covalently binds to FAD (Fig 1C). Depending upon different cyclopropylamines, several adducts were observed.[16, 17] Recently, we synthesized several known potent cyclopropylamine containing LSD1 inhibitors (e.g., compound **1**), which were tested for their activity against a panel of leukemia and solid tumors, showing potent in vitro and in vivo activity against several AML cell lines.[13] Given these promising antileukemia activity, more structure activity relationship (SAR) studies of LSD1 inhibitors are therefore needed. Here, we report synthesis, SAR and molecular modeling studies of a number of cyclopropylamine compounds, among which several cyclopropylimine compounds have been found to be a novel series of potent LSD1 inhibitors.

## Materials and methods

### Synthesis and characterization

All chemicals were purchased from Alfa Aesar (Ward Hill, MA) or Aldrich (Milwaukee, WI). ^1^H and ^13^C NMR spectra were used for compound identification on a Varian (Palo Alto, CA) 400-MR spectrometer. Purification of reaction products were carried out by silica gel (200–400 mesh) column chromatography monitored by UV at 254 nm. Analytical high performance liquid chromatography (HPLC) was performed on Shimadzu Prominence HPLC with a Zorbax C18 (or C8) column (4.6 x 250 mm) monitored by UV at 254 nm. The purities of the reported compounds were found to be >95%. The synthesis and characterization of compounds **1–40** can be found in Experimental Section.

### LSD1 enzyme inhibition

Human LSD1 catalytic domain, consisting of residues 172–833, was expressed in *Escherichia coli* BL21-CodonPlus strain (Agilent) as a GST fusion protein, by using a pGEX-KG vector. Briefly, the cells were grown to late log phase at 37°C and then were induced overnight with 0.2 mM IPTG at 25°C. Cells were harvested and lysed by French Press in PBS buffer and the supernatant was subjected to an affinity column chromatography using the glutathione sepharose resin. The eluted LSD1 fractions were further purified with ~90% purity by gel filtration on a Superdex 200 column (GE healthcare).

Horseradish peroxidase (HRP)-coupled assay was used for the evaluation of the inhibitors, based on the reaction rate (i.e., amount of the product H_2_O_2_) being quantitatively determined by adding HRP and a HRP fluorescence substrate Amplex red.[20] 30 nM LSD1 was incubated with increasing concentrations of a compound in 50 mM phosphate buffer (pH = 7.0 containing 0.01% Brij-35) for 30 min at 25°C. 10 μM of dimethylated peptide substrate ARTK(Me2)QTARKSTGGKAPRKQKA (*K*_m_~10 μM) was added to initiate the reaction. The final volume of the reaction is 60 μL. Upon 20 min incubation, 60 μL solution containing HRP (0.01 unit) and Amplex red (80 μM) was added to quench the reaction. The fluorescence of each well was then determined using a Beckman DTX-880 microplate reader (excitation at 535; emission at 595 nm). Data were imported into Prism 5.0 (GraphPad) and the IC_50_ values were calculated by using the sigmoidal dose response curve fitting in the software. The reported IC_50_s were the mean values of at least three independent experiments.

### Inhibition of MAO-A/-B

Inhibition of MAO-A and -B was determined using MAO-Glo assay kit (Promega). Following the manufacturer’s protocol, assays were performed in 384 well white plates (Corning) in the presence of 100 nM MAO-A/-B (Sigma) and MAO substrate. The final volume of each reaction mixture was 20 μL. Reactions were quenched after 60 min by adding reconstituted luciferin detection reagent (20 μL/well). 20 min after addition of detection reagent, the plate was measured in luminescence mode using Beckman DTX-880 microplate reader. IC_50_ calculation was done similarly.

### Docking

Docking studies were performed using our previous published methods [26] using Schrödinger suite (version 2016),[27] which includes all of the programs described below. The structures of LSD1 and MAO-A were prepared using the module “protein preparation wizard” in Maestro with default protein parameters. Hydrogen atoms were added, the ligand and all water molecules were removed, and FAD was retained in the protein structure for docking. Next, H-bonds were optimized, the partial charges for all atoms were assigned, and the protein-FAD complex was energy-minimized using OPLS-2005 force field. A receptor grid, which is large enough to contain the active site, was produced using the program Glide without any constraints. Inhibitor compound **10** were built, energy-minimized using OPLS-2005 force field in Maestro and then docked into the prepared protein structure using Glide (docking parameters: standard-precision and dock flexibly).

## Results and discussion

### Synthesis

**Fig 2:**
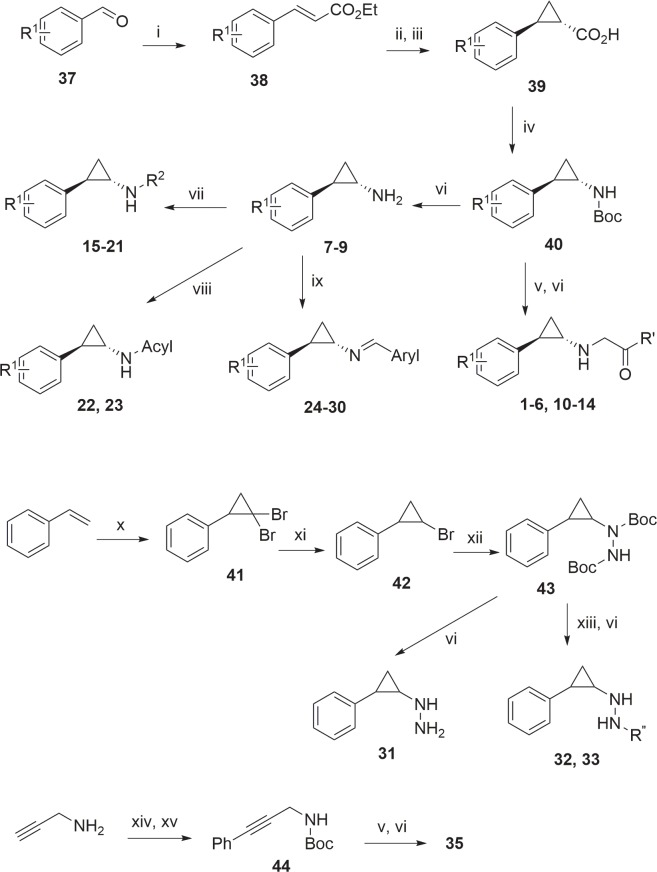
Reagents and conditions: (i) triethyl phosphonoacetate/*t*-BuOK, THF; (ii) Me_3_S(O)I, *t*-BuOK, DMSO; (iii) K_2_CO_3_, MeOH, reflux; (iv) diphenylphosphoryl azide, Et_3_N, *t*-BuOH, toluene, reflux; (v) NaH, 1-chloroacetyl-4-methylpiperazine, DMF; (vi) HCl/MeOH; (vii) aldehyde, NaBH_3_(CN), AcOH; (viii) Ac_2_O for compound 22 or PhNCO for 23; (ix) Aryl-CHO; (x) CHBr_3_, KOH; (xi) MeMgBr, Ti(O-*i*-Pr)_4_; (xii) Mg, then Boc-N = N-Boc; (xiii) NaH, then 1-chloroacetyl-4-(Boc)piperazine for 32 or 1-chloroformyl-4-(Boc)piperazine for 33; (xiv) (Boc)_2_O, Et_3_N; (xv) PhBr, CuI, Pd(dppf)Cl_2_, (*i*-Pr)_2_NEt.

The general methods for synthesizing compounds **1**–**35** are shown in Fig 2. The key reactions for making cyclopropylamine compounds **1**–**30** are the formation of trans-cyclopropyl ring and a Curtius rearrangement to give trans-cyclopropylamine.[20] Thus, a benzaldehyde **37** was reacted with sodium triethylphosphonoacetate to produce α, β-unsaturated ester **38**, which added to a methylene ylide produced *in situ* by trimethylsulfoxonium iodide and potassium *tert*-butoxide, giving trans-cyclopropyl carboxylic acid **39** ethyl ester. Upon hydrolysis, **39** was refluxed with diphenylphosphoryl azide and Et_3_N in toluene in the presence of anhydrous *tert*-BuOH. The one-pot reactions included the formation of cyclopropyl carbonyl azide and Curtius rearrangement to cyclopropyl isocyanate, which further reacted with *tert*-BuOH to produce *tert*-Butyloxycarbonyl (Boc) protected cyclopropylamine **40** in 55–70% yield. Alkylation of **40** followed by deprotection of Boc gave compounds **1**–**6** and **10**–**14**. Removal of Boc in **40** afforded cyclopropylamines **7**–**9**, which underwent reductive amination with an aldehyde to yield compounds **15**–**21**. These cyclopropylamines can be acylated to give compounds **22** and **23**, or reacted with an aryl carbaldehyde to produce aryl imine compounds **24**–**30**.

Synthesis of cyclopropyl hydrazine compounds is also shown in Fig 2. Styrene was reacted with bromoform and potassium hydroxide to form dibromocyclopropane **41**, which was treated with MeMgBr to remove one bromine atom, giving a ~6:1 mixture of trans- (major) and cis-cyclopropyl bromide **42**. Upon treatment of **42** with Mg, the cyclopropyl Grignard reagent thus obtained was added to di-*tert*-butyl azodicarboxylate (Boc-N = N-Boc) to produce the key intermediate **43**. Similar transformations of **43** can afford cyclopropyl hydrazine compounds **31**–**33** as a ~6:1 trans- and cis-mixture. Given the relatively low inhibitory activities of these compounds against LSD1 (Table 1), no isomer separation was performed. Propargylamine was protected with Boc and underwent a Sonogashira coupling to give compound **44**, which was similarly alkylated with 1-chloroacetyl-4-methylpiperazine, followed by deprotection, to produce compound **35**.

**Table 1:** Structures and LSD1 inhibitory activities of compounds 1–35.

	R^1^	R^2^	LSD1 IC_50_ (μM)
**1**	4-OBn	(4-methylpiperazin-1-yl)-carbonylmethyl	0.0098
**2**	4-(6-F-pyridin-3-yl)	same as above	0.077
**3**	4-Br	same as above	0.035
**4**	4-(6-Cl-pyridin-3-yl)	same as above	0.15
**5**	2-Br	same as above	>100
**6**	3-Br	same as above	>100
**7**	4-OBn	-H	0.91
**8**	6-F-pyridin-3-yl	-H	5.3
**9**	4-Br	-H	25
**10**	4-Br	(piperazin-1-yl)-carbonylmethyl	0.064
**11**	4-Br	(piperidin-1-yl)-carbonylmethyl	0.62
**12**	4-Br	(pyrrolidin-1-yl)-carbonylmethyl	3.6
**13**	4-H	same as **10**	0.19
**14**	4-H	same as **11**	1.3
**15**	4-Br	(piperidin-4-yl)-methyl	0.26
**16**	4-H	same as above	0.062
**17**	4-Br	-Bn	5.0
**18**	4-Br	-(4-OH)-Bn	94
**19**	4-Br	-(4-NH_2_)-Bn	>100
**20**	4-Br	-(4-NMe_2_)-Bn	71
**21**	4-Br	(pyridin-4-yl)-methyl	>100
**22**	4-Br	-COCH_3_	15.4
**23**	4-Br	-CONHPh	7.4
**24**	4-Br	= CH-Ph	15.6
**25**	4-Br	= CH-(4-Br-Ph)	1.2
**26**	4-Br	= CH-(2-OMe-Ph)	0.74
**27**	4-Br	= CH-(3-OMe-Ph)	0.90
**28**	4-Br	= CH-(2,3,4-OMe-Ph)	94
**29**	4-Br	= CH-(4-NMe_2_-Ph)	11
**30**	4-Br	= CH-(pyridine-4-yl)	14
**31**	4-H	-NH_2_	5.8
**32**	4-H	(piperazin-1-yl)-carbonylmethylamino	0.67
**33**	4-H	(piperazin-1-yl)-carboxamido	84
**34**	4-Br	(piperidin-4-yl)-methylamino	1.7
**35**	1-(4-methylpiperazin-1-yl)-2-((3-phenylprop-2-yn-1-yl)amino)ethan-1-one		36.4

### Structure activity relationships (SAR)

Table 1 (or S1 Table with embedded structures) summarizes the structure activity relationships of compounds 1–35. First, we found known LSD1 inhibitors **1**–**3** showed potent inhibitory activity against recombinant human LSD1 with IC_50_ values of 9.8, 77 and 35 nM (Table 1), respectively. These results are consistent with previously reported activities[20] and show our biochemical assay is robust. In addition, the activities of **1**–**3** suggest that LSD1 can well accommodate a wide range of para-substituted R^1^ groups ranging from a large, flexible benzyloxy, a rigid pyridinyl, to a smaller -Br, which upon completion of LSD1 catalyzed reaction, bind covalently to the FAD moiety. Compound **4** (IC_50_ = 0.15 μM) having a 6-Cl-pyridin-3-yl R^1^ group is slightly less active than its F analog **2**. However, compounds **5** and **6** with a 2- and 3-bromo substituent, which are close analogs of **3** bearing a 4-Br, were found to be essentially inactive against LSD1 (IC_50_ > 100 μM). These results, together with the potent activities of compounds **1**–**4**, suggest that substitution at the *ortho*- or *meta*-position of the phenyl group is highly disfavored, while a broad range of R^1^ groups at the *para*-position may be favorable.

Next, compounds **7**–**9**, which do not have the (4-methylpiperazin-1-yl)carbonylmethyl (abbreviated as 4-methyl-PCM thereafter) R^2^ substituent, were synthesized and found to inhibit LSD1 with IC_50_ values of 0.91, 5.3 and 25 μM, respectively. They are 92-, 69- and 714-fold less active than the corresponding compounds **1**–**3**, showing the 4-methyl-PCM group is critically important for potent LSD1 inhibition. While compound **10** (IC_50_ = 0.064 μM) with a PCM group exhibited a comparable activity to that of **3**, compounds **11** and **12** having a (piperidin-1-yl)carbonylmethyl and (pyrrolidin-1-yl)carbonylmethyl substituent are ~10 and 56× less active than **10**. Thus, the activities of compounds **3**, **10**–**12** suggest an important role of the terminal basic amine moiety in PCM of compounds **3** and **10**. This is further confirmed by the inhibitory activities of de-bromo compounds **13** and **14**: compound **13** (IC_50_ = 0.19 μM) with the PCM group is ~7-fold more active than **14** (IC_50_ = 1.3 μM) without a terminal amine group.

Compounds **15** and **16** containing a piperidin-4-ylmethyl R^2^ substituent are also potent LSD1 inhibitors with their IC_50_ values of 260 and 62 nM. Analogous compound **17** having a benzyl R^2^ substituent is significantly less active (IC_50_ = 5 μM), showing again the importance of a basic amine group at the terminal of -R^2^. Compounds **18**–**21** with a 4-OH-, 4-NH_2_-, 4-NMe_2_-benzyl and 4-pyridinylmethyl, respectively, were synthesized in order to find if any of these R^2^ groups can increase the inhibitory activity. However, as compared to the activity of compound **17**, none of these groups are favorable (IC_50_: 71 - >100 μM). Possibly, the reduced basicity of the aniline/pyridine groups and/or the aromatic rings could be responsible for the significant activity decreases. Compounds **22** and **23** with an electron-withdrawing acetyl and phenylaminocarbonyl R^2^ group, respectively, exhibited moderate activities against LSD1 with the IC_50_ values of 15.4 and 7.4 μM. Their activities are comparable to those of compounds **9** (R^2^ = H) and **17** (R^2^ = Bn). However, these compounds are several orders of magnitude less active than those bearing a basic PCM group (e.g., **3**, **10**, **13**, **15** and **16**).

A series of cyclopropylimine compounds **24**–**30** were readily synthesized by a one-step reaction of trans-(4-bromophenyl)-cyclopropylamine with an aromatic aldehyde. Compound **24** prepared from benzaldehyde exhibited a modest activity (IC_50_ = 15.6 μM). However, Compounds **25**–**27** with a 4-Br, 2-OMe and 3-OMe substituent are >10× more active, with IC_50_ of 1.2, 0.74 and 0.9 μM, respectively. 3,4,5-OMe substituted compound **28** almost loses the inhibitory activity. Compounds **29** and **30** having a 4-NMe_2_-phenyl or 4-pyridinyl group possess only modest activities. Given the strong LSD1 inhibitory activities as well as novel structures of compounds **25**–**27**, more SAR studies may be needed as a future direction for inhibitor development.

Since hydrazine and propargylamine (Fig 1B) compounds were reported to be LSD1 inhibitors,^17^ compounds **31**–**35** were synthesized and tested for their activity against LSD1. Trans-phenyl cyclopropyl hydrazine (**31**) was found to be a moderate LSD1 inhibitor with an IC_50_ of 5.8 μM. Adding a PCM group in **32** resulted in ~9-fold enhanced activity (IC_50_ = 0.67 μM). However, **32** is still ~3× less active than its cyclopropylamine analog **13**. Replacing the PCM with a (piperazin-1-yl)carbonyl group in compound **33** (IC_50_ = 84 μM), which has almost the same molecular length as that of **13**, led to a complete loss of LSD1 inhibition. Compound **34** (IC_50_ = 1.7 μM) is also significantly (~7×) less active than its cyclopropylamine analog **15**. The propargylamine analog **35** having a 4-methyl-PCM substituent has only weak activity against LSD1 (IC_50_ = 36.4 μM). These results show that cyclopropylamine is a superior core structure to cyclopropyl hydrazine and propargylamine.

### Activity against MAO-A/-B and selectivity for LSD1

Because the cyclopropylamine compounds were derived from MAO-A/-B inhibitor tranylcypromine, we next tested 11 selected compounds for their activity against human MAO-A and -B and the results are summarized in Table 2. 4-Methyl-PCM containing compounds **1**–**3** have only modest activity (IC_50_: 7.3–480 μM) against MAO-A and -B, showing excellent selectivity of 200->1,000-folds for LSD1 inhibition. These IC_50_ values are larger than those in a previous report,[20] presumably due to different assay conditions. However, these differences do not affect the conclusion that **1**–**3** are highly selective LSD1 inhibitors. Compounds **7** and **8** without 4-methyl-PCM exhibited more potent inhibition against MAO-A and -B (IC_50_: 0.17–3.1 μM) than against LSD1. Similarly to **1**–**3**, potent LSD1 inhibitor **10** having a PCM substituent is a weak inhibitor of MAO-A and–B and shows a high selectivity of >280-folds. Of interest are the MAO-A/-B activity and selectivity for compound **11**: a single change from -NMe- (or -NH-) in compound **3** (or **10**) to -CH_2_- resulted in more inhibitory activity for **11** against MAO-A and -B (IC_50_: 0.26 and 0.51 μM). Potent LSD1 inhibitor **16** (IC_50_: 62 nM) with a basic piperidin-4-ylmethyl R^2^ group was against found to exhibit relatively weak activity against MAO-A and -B (IC_50_: 2.4 and 1.8 μM), although the selectivity is diminished to ~30-fold. These results suggest a basic PCM or related group (e.g., that in **16**) is needed to provide both high potency and selectivity for LSD1 inhibition. Similar selectivity profiles can be found for compounds **26** and **32**: no LSD1 selectivity was observed for cyclopropylimine **26** with a neutral R^2^ group, while cyclopropylhydrazine **32** bearing a PCM group exhibited >150× selectivity for LSD1 inhibition. Its parent compound **31** (without a R^2^ group) exhibited modest activity against MAO-A and -B (IC_50_: 40.7 and 87 μM).

**Table 2:** Inhibitory activity (IC_50_, μM) against MAO-A and -B and selectivity index for LSD1.

	LSD1	MAO-A	MAO-B	Selectivity index
**1**	0.0098	17.5	34.2	>1,700
**2**	0.077	120	480	>1,500
**3**	0.035	7.3	16.3	>208
**7**	0.91	0.17	0.35	<0.38
**8**	5.3	0.42	3.1	<0.58
**10**	0.064	18.1	23.1	>280
**11**	0.62	0.26	0.51	<0.82
**16**	0.062	2.4	1.8	>29
**26**	0.74	0.30	0.40	<0.54
**31**	5.8	40.7	87.0	>7
**32**	0.67	97.5	370	>145

### Molecular modeling studies

Based on the above SAR and structure selectivity relationship results, it is of great interest why the terminal basic amine containing R^2^ group in compounds **1**–**6**, **10**, **13** and **15** significantly increase inhibitory activity as well as selectivity for LSD1. These cyclopropylamine-containing compounds are not competitive inhibitors of LSD1. Rather, upon completion of the LSD1 catalyzed reaction, the R^1^-cyclopropyl moiety is covalently linked to FAD, while the R^2^-NH_2_ is eliminated from LSD1 (Fig 1C).[16, 17] Because of this mechanism of catalysis, X-ray crystallography is not suited for the investigation of the role of the R^2^ group, as only the R^1^ moiety could be visualized. From our experimental data, it is a reasonable hypothesis that the R^2^ terminal amine group plays a favorable role in the initial binding of the whole inhibitor molecule to LSD1, which occurs before FAD-mediated oxidation. However, the amine-containing R^2^ group does not help the initial binding of these compounds to MAO-A (or -B). The binding affinity as well as the conformation of the initial, non-covalent protein-inhibitor complex could affect the oxidation process and overall inhibitory activity. Docking was used to find possible evidence to support the hypothesis.

X-ray structures of human LSD1 and MAO-A in complex with FAD and a variety of inhibitors or substrate analogs have been reported and available in the RCSB Protein Data Bank. Molecular modeling was performed using Schrödinger Suite software package (Version 2016).[27] Tertiary structures of LSD1 (PDB: 2V1D)[28] and MAO-A (PDB: 2Z5X)[29] were used as docking templates. The proteins were prepared by removing their bound inhibitor or substrate, while keeping FAD as an integrate part of the proteins. A representative compound **10**, which exhibited potent LSD1 inhibition (IC_50_ = 64 nM) with excellent selectivity (>280-fold) against MAO-A, was selected as a model compound.

**Fig 3:**
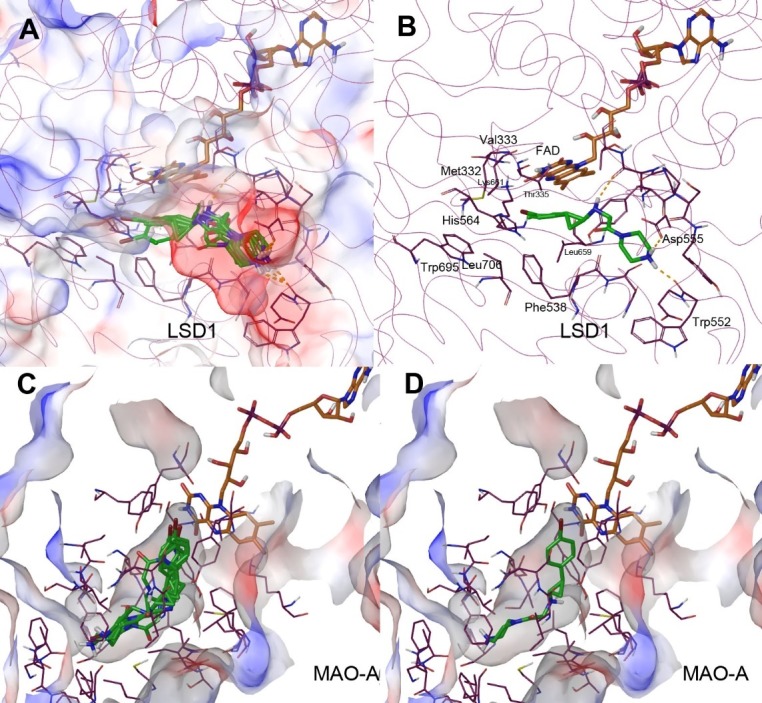
Docking results of compound 10 (as tube models with C atoms in green). (**A**) Five docking structure of **10** with the lowest energies in the active site of LSD1 (shown as a semi-transparent electrostatic surface); (**B**) The lowest-energy docking structure in the active site of LSD1, showing selected interacting amino acid residues (with C atoms in purple) and FAD (with C atoms in brown); (**C**) Five docking structure of **10** with the lowest energies in the active site of MAO-A (shown as a semi-transparent electrostatic surface); (**D**) The lowest-energy docking structure in the active site of MAO-A, showing selected interacting amino acid residues (with C atoms in purple) and FAD (with C atoms in brown).

LSD1 has a very large substrate-binding pocket widely open to the solvent, able to accommodate >10 amino acid residues of histone peptide.[28] As shown in Fig 3A and 3B, compound **10** can be favorably docked into the LSD1-FAD structure, with its 5 docking structures with the lowest energies adopting a similar overall conformation. Particularly noteworthy is that the protonated R^2^ terminal amino groups (at the physiological *p*H) of all these structures form strong H-bond and electrostatic interactions with the sidechain of Asp555 and the carbonyl group of Trp552. Moreover, the α-C and N atoms of the cyclopropylamine moiety are located ~5Å from the flavin ring, in an orientation ready for oxidation. In addition, the 4-bromophenyl (R^1^) group of **10** is predicted to be located in and have favorable interactions with a mainly hydrophobic pocket surrounded by Met332, Val333, Thr335, Leu659, Lys661, Phe538, His564, Leu706 and Trp695. This binding model seems to be consistent with the experimental results and can explain the critical role of the terminal amino-containing R^2^ group in high affinity binding and potent inhibition of LSD1.

Next, we docked compound **10** into the structure of MAO-A. Drastically different from LSD1, MAO-A (as well as MAO-B) has a relatively small active site (or substrate binding pocket), which is deeply located in the center and not freely accessible from outside of the enzyme.[29] Entry of a substrate or inhibitor into MAO-A’s active site is controlled by the conformation of a flexible loop 108–118. Using a similar method, compound **10** can be docked into the active site of MAO-A, with its 5 lowest-energy docking structures nicely fitted inside the mostly hydrophobic active site (Fig 3C and 3D). However, the α-C and N atoms of the cyclopropylamine moiety of **10** are located ≥7.5 Å from the flavin ring. These binding models require additional conformational changes of the protein and/or the inhibitor for the ensuing oxidation to occur, which seem to be disfavored. In addition, the terminal positively charged amino group of R^2^ has no favorable interactions with the protein (Fig 3D). These modeling studies might rationalize the weak inhibitory activity of compound **10** against MAO-A (IC_50_ = 18.1 μM) as well as the high selectivity for LSD1.

We also performed docking studies of compound **26**, a cyclopropylimine compound that exhibited comparable inhibitory activity against LSD1 (IC_50_ = 740 nM) and MAO-A (IC_50_ = 300 nM). As shown in S1 Fig, compound **26** can be comfortably docked into the active sites of LSD1 and MAO-A, with both α-C atoms of the cyclopropylimine moiety located ~6 Å from the flavin ring. The docking results seem to be consistent with the inhibitory activities of compound **26**.

## Conclusion

Aberrant histone modifications are often found in many types of cancer. LSD1, a demethylase for H3K4-Me2 or -Me1, has recently been found to be a drug target for AML. More structure activity relationship studies targeting LSD1 are therefore needed. In this study, a total of 35 cyclopropylamine and related compounds were synthesized and tested for their activities against recombinant human LSD1. The enzyme selectivity of selected compounds were also evaluated using related MAO-A and -B. SARs for these compounds include 1) a PCM or related R^2^ group that contains a basic amine functionality is critically important for both potent LSD1 inhibition and high selectivity; 2) while an *ortho*- or a *meta*-R^1^ substitution in the phenylcyclopropylamine is disfavored, the *para*-position may tolerate a broad range of substituents for potent LSD1 inhibition; 3) cyclopropylamine seems to be the best core structure for LSD1 inhibition, as compared to hydrazines and propargylamines; and 4) a novel series of cyclopropylimine compounds were found to be submicromolar inhibitors of LSD1. Docking studies were performed and provided possible binding models of compound **10** in LSD1 and MAO-A. Moreover, these modeling studies can rationalize the observed SARs and selectivity, particularly with respect to the critical role of the terminal basic amine group of R^2^ of these cyclopropylamine-containing compounds.

## Supporting information

S1 Fig Docking results of compound 26 (as tube models with C atoms in green). (A) The lowest-energy docking structure in the active site of LSD1; and (B) The lowest-energy docking structure in the active site of MAO-A, showing selected interacting amino acid residues (with C atoms in purple) and FAD (with C atoms in brown).
(PDF)

S1 File Experimental section. Detailed compound synthesis and characterization.
(PDF)

S1 Table Structures and LSD1 inhibitory activities of compounds 1–35 with embedded structures. (PDF)

## References

[1] Kouzarides T. Cell 2007, 128, 693 10.1016/j.cell.2007.02.005 17320507

[2] Jones P. A.; Baylin S. B. Cell, 2007, 128, 683 10.1016/j.cell.2007.01.029 17320506 PMC3894624

[3] Minucci S; Pelicci P. G. Nat. Rev. Cancer 2006, 6, 38 10.1038/nrc1779 16397526

[4] Copeland R. A.; Solomon M. E.; Richon V. M. Nat. rev. Drug Discov. 2009, 8, 724 10.1038/nrd2974 19721445

[5] Shi Y.; Lan F.; Matson C.; Mulligan P.; Whetstine J. R.; Cole P. A.; Casero R. A. Cell 2004, 119, 941 10.1016/j.cell.2004.12.012 15620353

[6] Metzger E.; Wissmann M.; Yin N.; Müller J. M.; Schneider R.; Peters A. H.; Günther T.; Buettner R.; Schüle R. Nature 2005, 437, 436 10.1038/nature04020 16079795

[7] Wang J.; Hevi S.; Kurash J. K.; Lei H.; Gay F.; Bajko J.; Su H.; Sun W.; Chang H.; Xu G.; Gaudet F.; Li E.; Chen T. Nat. Genet. 2009, 41, 125 10.1038/ng.268 19098913

[8] Kerenyi M. A.; Shao Z.; Hsu Y. J.; Guo G.; Luc S.; O'Brien K.; Fujiwara Y.; Peng C.; Nguyen M.; Orkin S. H. Elife 2013, 2, e00633 10.7554/eLife.00633 23795291 PMC3687337

[9] Kahl P.; Gullotti L.; Heukamp L. C.; Wolf S.; Friedrichs N.; Vorreuther R.; Solleder G.; Bastian P. J.; Ellinger J.; Metzger E.; Schule R.; Buettner R. Cancer Res. 2006, 66, 11341. 17145880 10.1158/0008-5472.CAN-06-1570

[10] Schulte J. H.; Lim S.; Schramm A.; Friedrichs N.; Koster J.; Versteeg R.; Ora I.; Pajtler K.; Klein-Hitpass L.; Kuhfittig-Kulle S.; Metzger E.; Schule R.; Eggert A.; Buettner R.; Kirfel J. Cancer Res. 2009, 69, 2065. 19223552 10.1158/0008-5472.CAN-08-1735

[11] Lim S.; Janzer A.; Becker A.; Zimmer A.; Schule R.; Buettner R.; Kirfel J. Carcinogenesis 2010, 31, 512 10.1093/carcin/bgp324 20042638

[12] Harris W. J.; Huang X.; Lynch J. T.; Spencer G. J.; Hitchin J. R.; Li Y.; Ciceri F.; Blaser J. G.; Greystoke B. F.; Jordan A. M.; Miller C. J.; Ogilvie D. J.; Somervaille T. C. Cancer Cell 2012, 21, 473 10.1016/j.ccr.2012.03.014 22464800

[13] Feng Z.; Zhou C.; Wu F.; Yao Y.; Wei L.; Liu W.; Dong S.; Redell M.; Song Y. J. Hematol. Oncol. 2016, 9, 24. 10.1186/s13045-016-0252-7 PMC4789278 26970896

[14] Song Y.; Wu F.; Wu J. J. Hematol. Oncol. 2016, 9, 49. 10.1186/s13045-016-0279-9 PMC4912745 27316347

[15] http://www.lls.org/#/diseaseinformation/leukemia/.

[16] Binda C.; Valente S.; Romanenghi M.; Pilotto S.; Cirilli R.; Karytinos A.; Ciossani G.; Botrugno O. A.; Forneris F.; Tardugno M.; Edmondson D. E.; Minucci S.; Mattevi A.; Mai A. J. Am. Chem. Soc. 2010, 132, 6827. 10.1021/ja101557k 20415477

[17] Mohammad H. P.; Smitheman K. N.; Kamat C. D.; Soong D.; Federowicz K. E.; Van Aller G. S.; Schneck J. L.; Carson J. D.; Liu Y.; Butticello M.; Bonnette W. G.; Gorman S. A.; Degenhardt Y.; Bai Y.; McCabe M. T.; Pappalardi M. B.; Kasparec J.; Tian X.; McNulty K. C.; Rouse M.; McDevitt P.; Ho T.; Crouthamel M.; Hart T. K.; Concha N. O.; McHugh C. F.; Miller W. H.; Dhanak D.; Tummino P. J.; Carpenter C. L.; Johnson N. W.; Hann C. L.; Kruger R. G. A DNA Hypomethylation Signature Predicts Antitumor Activity of LSD1 Inhibitors in SCLC. Cancer Cell 2015, 13, 57. 10.1016/j.ccell.2015.06.002 26175415

[18] See recent reviews (Ref. 18 and 19) and the references therein: Suzuki, T.; Miyata, N. J. Med. Chem. 2011, 54, 8236.

[19] Mould D. P.; McGonagle A. E.; Wiseman D. H.; Williams E. L.; Jordan A. M. Med. Res. Rev. 2015, 35, 586 10.1002/med.21334 25418875

[20] For more recent LSD1 inhibitors, see Ref. 20–26: Neelamegam, R.; Ricq, E. L.; Malvaez, M.; Patnaik, D.; Norton, S.; Carlin, S. M.; Hill, I. T.; Wood, M. A.; Haggarty, S. J.; Hooker, J. M. ACS Chem. Neurosci. 2012, 3, 120. 10.1021/cn200104y PMC3382965 22754608

[21] Zheng Y. C.; Duan Y. C.; Ma J. L.; Xu R. M.; Zi X.; Lv W. L.; Wang M. M.; Ye X. W.; Zhu S.; Mobley D.; Zhu Y. Y.; Wang J. W.; Li J. F.; Wang Z. R.; Zhao W.; Liu H. M. J. Med. Chem. 2013, 56, 8543 10.1021/jm401002r 24131029 PMC3881423

[22] Sorna V.; Theisen E. R.; Stephens B.; Warner S. L.; Bearss D. J.; Vankayalapati H.; Sharma S. J. Med. Chem. 2013, 56, 9496. 10.1021/jm400870h 24237195

[23] Prusevich P.; Kalin J. H.; Ming S. A.; Basso M.; Givens J.; Li X.; Hu J.; Taylor M. S.; Cieniewicz A. M.; Hsiao P. Y.; Huang R.; Roberson H.; Adejola N.; Avery L. B.; Casero R. A. Jr.; Taverna S. D.; Qian J.; Tackett A. J.; Ratan R. R.; McDonald O. G.; Feinberg A. P.; Cole P. A. ACS Chem. Biol. 2014, 9, 1284 10.1021/cb500018s 24707965 PMC4076021

[24] Kutz C. J.; Holshouser S. L.; Marrow E. A.; Woster P. M. MedChemComm 2014, 5, 1863 10.1039/C4MD00283K 25580204 PMC4286191

[25] Hitchin J. R.; Blagg J.; Burke R.; Burns S.; Cockerill M. J.; Fairweather E. E.; Hutton C.; Jordan A. M.; McAndrew C.; Mirza A.; Mould D.; Thomson G. T.; Waddell I.; Ogilvie D. J. MedChemComm. 2014, 4, 1513.

[26] Wu F.; Zhou C.; Yao Y.; Wei L.; Feng Z.; Deng L.; Song Y. J. Med. Chem. 2016, 59, 253. 10.1021/acs.jmedchem.5b01361 PMC4878443 26652247

[27] Schrödinger Suite, version 2016, Schrödinger, LLC, New York, NY, 2016.

[28] Forneris F.; Binda C.; Adamo A.; Battaglioli E.; Mattevi A. J. Biol. Chem. 2007, 282, 20070. 10.1074/jbc.C700100200 17537733

[29] Son S.Y.; Ma J.; Kondou Y.; Yoshimura M.; Yamashita E.; Tsukihara T. Proc. Natl. Acad. Sci. USA 2008, 105, 5739 10.1073/pnas.0710626105 18391214 PMC2311356

