# Supplementary material for: Structure activity relationship and modeling studies of inhibitors of lysine specific demethylase 1
Source: PLoS One. 2017 Feb 3;12(2):e0170301. doi: 10.1371/journal.pone.0170301 (PMC5291514; doi:10.1371/journal.pone.0170301)
Supplement: S1 Fig — (A) The lowest-energy docking structure in the active site of LSD1; and (B) The lowest-energy docking structure in the active site of MAO-A, showing selected interacting amino acid residues (with C atoms in purple) and FAD (with C atoms in brown). (PDF) [file pone.0170301.s001.pdf]

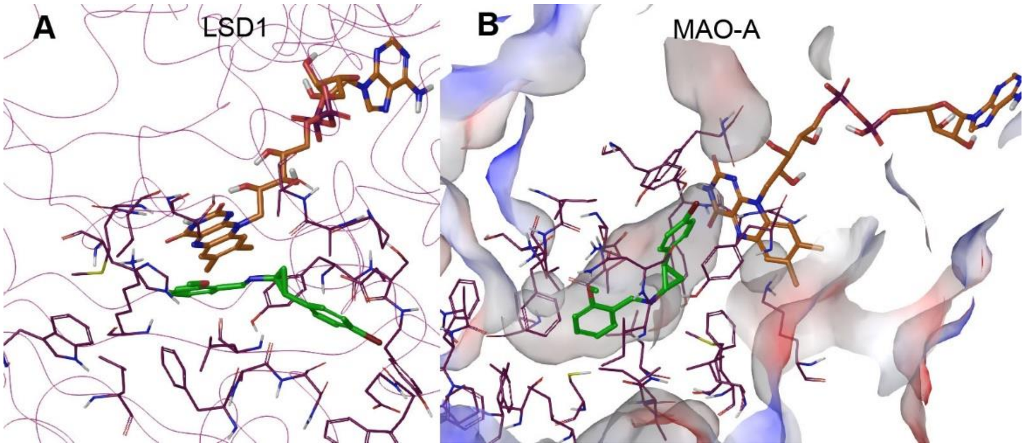

Figure S1. Docking results of compound **26** (as tube models with C atoms in green). (**A**) The lowest-energy docking structure in the active site of LSD1; and (**B**) The lowest-energy docking structure in the active site of MAO-A, showing selected interacting amino acid residues (with C atoms in purple) and FAD (with C atoms in brown).
