# Supplementary material for: Structure activity relationship and modeling studies of inhibitors of lysine specific demethylase 1
Source: PLoS One. 2017 Feb 3;12(2):e0170301. doi: 10.1371/journal.pone.0170301 (PMC5291514; doi:10.1371/journal.pone.0170301)
Supplement: S1 File — Detailed compound synthesis and characterization. (PDF) [file pone.0170301.s002.pdf]

Structure Activity Relationship and Modeling Studies of Inhibitors of Lysine Specific Demethylase 1

Chao Zhou[¶]*, Fangrui Wu*[¶]*, Lianghao Lu, Liping Wei, Eric Pai, Yuan Yao, and Yongcheng Song**

Department of Pharmacology, Baylor College of Medicine, 1 Baylor Plaza, Houston, TX 77030, USA.

Experimental Section

All chemicals for synthesis were purchased from Alfa Aesar (Ward Hill, MA) or Aldrich (Milwaukee, WI). The compound identity was characterized by ^{1}H NMR on a Varian (Palo Alto, CA) 400-MR spectrometer. The purities of synthesized compounds were determined by a Shimadzu Prominence HPLC with a Zorbax C18 (or C8) column (4.6 x 250 mm) monitored by UV at 254 nm. The purities of the reported compounds were found to be >95%.

General method A. Triethyl phosphonoacetate (13.5 g, 60 mmol) was added dropwise to a suspension of *t*-BuOK (6.7 g, 60 mmol) in anhydrous THF at -5 ºC under N_2 protection, after which an aldehyde **37** (e.g., 4-bromobenzaldehyde, R^1 = 4-Br, 9.3 g, 50 mmol) was added. The mixture was stirred for 5 h and quenched with water (1000 mL). The product was extracted with ethyl acetate (3 x 300 mL) and the combined organic layers were washed with saturated $NaHCO_3$ solution and brine, dried over Na_2SO_4. Removal of solvent yielded crude compound **38** as a pale yellow oil (12.2 g, 96% yield), which is pure enough for use in the next step. ^{1}H NMR (400 MHz, $CDCl_3$): δ 7.60 (d, J = 16.0 Hz, 1H), 7.51 (d, J = 8.4

Hz, 2H), 7.38 (d, J = 8.4 Hz, 2H), 6.41 (d, J = 16.0 Hz, 1H), 4.26 (q, J = 7.2 Hz, 2H), 7.51 (t, J = 7.2 Hz, 3H).

To a suspension of NaH (60% in oil, 1.0 g, 24 mmol) in anhydrous DMSO (80 mL), trimethyl-sulfoxonium iodide (5.3 g, 24 mmol) was added in 5 portions in 15 min, yielding a clear solution after stirring for 1 h. Compound **38** (5.1 g, 20 mmol) was then added dropwise. The mixture was stirred for 4 h and quenched with water (200 mL). The product was extracted with ethyl acetate (3 x 150 mL) and the combined organic layers were washed with saturated $NaHCO_3$ solution and brine, dried over Na_2SO_4. Removal of solvent yielded the ethyl ester of **39** (4.5 g, 84 % yield). which is pure enough for next step. ^{1}H NMR (400 MHz, $CDCl_3$): δ 7.39 (d, J = 8.4 Hz, 2H), 6.97 (d, J = 8.4 Hz, 2H), 4.16 (q, J = 7.2 Hz, 2H), 2.50-2.43 (m, 1H), 1.89-1.83 (m, 1H), 1.62-1.56 (m, 1H), 1.27 (t, J = 7.2 Hz, 3H), 0.91-0.79 (m, 1H).

To a solution of compound **39** (2.7 g, 10 mmol) in EtOH (40 mL), 40 mL of 1N LiOH was added dropwise and the resulting solution was stirred for 5 h. Upon removal of EtOH under reduced pressure, the aqueous solution was washed with 10 mL of ether, acidified with 1 N HCl to *p*H 2, extracted with ethyl acetate (4 x 50 mL). The organic phases were washed with brine, dried over Na_2SO_4, and concentrated. Recrystallization of the residue with ethyl acetate and *n*-hexane gave compound **39** (1.5 g, 68% yield). ^{1}H NMR (400 MHz, $CDCl_3$): δ 7.40 (d, J = 8.4 Hz, 2H), 6.98 (d, J = 8.4 Hz, 2H), 2.60-2.51 (m, 1H), 1.95-1.83 (m, 1H), 1.71-1.62 (m, 1H), 1.40-1.32 (m, 1H).

A mixture of compound **39** (4.8 g, 20 mmol), diphenylphosphoryl azide (5.5 g, 20 mmol), anhydrous Et_3N (4.0 g, 40 mmol) in anhydrous *t*-BuOH (200 mL) were heated to 90 °C and stirred for 18 h. Upon removal of solvents, the residue was dissolved in 200 mL of ethyl acetate, washed with brine, dried over Na_2SO_4, and concentrated. Column chromatography (silica gel, ethyl acetate: *n*-hexane from 1:40 to 1:5) gave compound **40** as a pale yellow oil (4.2 g, 64% yield). ^{1}H NMR (400 MHz, $CDCl_3$): δ 7.37 (d, J = 8.4 Hz, 2H), 7.01 (d, J = 8.4 Hz, 2H), 4.95-4.66 (bs, 1H), 2.71-2.60 (m, 1H), 2.09-1.93 (m, 1H), 1.45 (s, 9H), 1.19-1.06 (m, 2H).

General method B. Compound **40** (R^1 = 4-(6-F-pyridin-3-yl), 2.4 g, 7.3 mmol) was added to a suspension of NaH (0.6 g, 14.6 mmol) in 20 mL of anhydrous DMF and the resulting mixture stirred at 0 ºC for 1 h. 1-Chloroacetyl-4-methylpiperazine (as a hydrochloric acid salt, 1.6 g, 7.4 mmol) (or another alkylating reagent) was added and stirring was continued for 1 h at 0 ºC. Upon completion, the reaction was quenched with water and the product was extracted with ethyl acetate (3 x 30 mL). The combined organic layers were washed with brine (30 mL), dried over Na_2SO_4, and evaporated. Column chromatography (silica gel, CH_2Cl_2: MeOH from 40:1 to 10:1) gave the Boc-protected final product **2** as a white solid (1.8 g, 53% yield). ^1H NMR (400 MHz, $CDCl_3$): δ 8.36 (s, 1H), 7.92 (dt, *J* = 7.6 & 1.6 Hz, 1H), 7.41 (d, *J* = 8.4 Hz, 2H), 7.25-7.11 (br, 2H), 6.97 (dd, *J* = 8.4 & 3.2 Hz, 1H), 4.22-4.11 (m, 1H), 3.72-3.56 (m, 2H), 3.49-3.40 (m, 2H), 3.12-2.98 (m, 1H), 2.46-2.31 (m, 4H), 2.29-2.19 (m, 3H), 2.07-1.92 (m, 2H), 1.39 (s, 9H), 1.35-1.06 (m, 2H).

Deprotection of the above compound (1.8 g, 3.9 mmol) was performed in 0.6 N HCl in MeOH (55 mL) at 0 ºC under N_2 protection for 1 h. Upon removal of the solvents under reduced pressure, 30 mL of anhydrous ether was added to the residue with stirring and the solid was filtered, washed with anhydrous ether twice (20 mL for each) and dried under high vacuum to give dihydrochloric acid salt of compound **2** as a white solid (1.15 g, 62 % yield).

General method C: Boc-deprotection of compound **40** as described above yielded the hydrochloric acid salt of primary amine compounds **7-9** as white powder.

To make compounds **15-21**, a mixture of a primary amine compound, such as **9** (90 mg, 0.31 mmol), an aldehyde (0.33 mmol), acetic acid (0.209 mL) and $NaBH_3CN$ (20 mg, 0.32 mmol) in methanol (12 mL) was stirred overnight. The solution was neutralized with saturated $NaHCO_3$ to *p*H 8 and extracted with ethyl acetate (3 x 50 mL). The organic layers were dried over Na_2SO_4 and concentrated. Column chromagraphy (silica gel, EtOAc: *n*-hexane from 1:20 to 1:5) afforded the alkylated products.

To make imine compounds **24-30**, a mixture of a primary amine compound, such as **9** (150 mg, 0.5 mmol), an aldehyde (0.5 mmol) and anhydrous $MgSO_4$ (600 mg, 5.0 mmol) in anhydrous CH_2Cl_2 (5 mL) was stirred for 18 h. $MgSO_4$ was filtered off and the filtrate was concentrated to give the imine product as a yellow oil in almost quantitative yield.

Compound 1. It was prepared from 4-benzyloxy-benzaldehyde and 1-chloroacetyl-4-methylpiperazine, following the general methods A and B, as a dihydrochloric acid salt (white powder). ^{1}H NMR (400 MHz, DMSO-d^{6}): δ 11.81-11.42 (m, 1H), 9.88-9.54 (m, 1H), 7.49-7.30 (m, 5H), 7.11-6.86 (m, 4H), 5.06 (d, *J* = 10.4 Hz, 2H), 4.48-3.81 (m, 4H), 3.48-3.35 (m, 2H), 3.34-2.86 (m, 4H), 2.74 (s, 3H), 2.59-2.52 (m, 1H), 2.13-2.00 (m, 1H), 1.59-1.50 (m, 1H), 1.21-1.08 (m, 1H); ^{13}C NMR (100 MHz, DMSO-d^{6}): 165.1, 153.5, 136.7, 132.1, 128.0, 127.1, 126.8, 126.2, 111.7, 70.8, 52.7, 49.2, 48.3, 45.6, 36.1, 23.9, 15.3; MS (ESI) $[M+H]^+$ 380.2.

Compound 2. It was prepared from 4-(6-fluoropyridin-3-yl)-benzaldehyde and 1-chloroacetyl-4-methylpiperazine, following general methods A and B, as a dihydrochloric acid salt (white powder). ^{1}H NMR (400 MHz, DMSO-d6): δ 8.27 (s, 1H), 8.07 (t, *J* = 8.0 Hz, 1H), 7.53 (d, *J* = 7.2 Hz, 2H), 7.22 (d, *J* = 7.2 Hz, 2H), 7.07 (d, *J* = 8.0 Hz, 1H), 4.63 (d, *J* = 8.4 Hz, 1H), 4.51-4.41 (m, 1H), 4.33-4.22 (m, 1H), 3.93-3.82 (m, 1H), 3.65-3.57 (m, 4H), 3.11-2.98 (m, 4H), 2.83 (s, 3H), 1.55-1.50 (m, 1H), 1.41-1.32 (m, 1H); ^{13}C NMR (100 MHz, DMSO-d^{6}): 165.3, 157.5, 141.3, 140.7, 133.6, 132.5, 127.0, 112.9, 52.0, 48.5, 48.3, 45.1, 36.8, 23.7, 15.9; MS (ESI) $[M+H]^+$ 369.2.

Compound 3. It was prepared from 4-bromobenzaldehyde and 1-chloroacetyl-4-methylpiperazine, following general methods A and B, as a dihydrochloric acid salt (white powder). ^{1}H NMR (400 MHz, DMSO-d6): δ 11.61-11.50 (bs, 1H), 9.88-9.59 (bs, 1H), 7.49 (d, *J* = 8.4 Hz, 2H), 7.16 (d, *J* = 8.4 Hz, 2H), 4.38-4.20 (m, 2H), 3.95-3.81 (m, 1H), 3.66-3.03 (m, 8H), 2.76 (s, 3H), 2.59-2.52 (m, 1H), 1.62-1.57 (m, 1H), 1.29-1.24 (m, 1H); ^{13}C NMR (100 MHz, DMSO-d^{6}): 164.8, 138.4, 130.5, 126.8, 119.9, 51.5, 49.3, 48.6, 46.6, 36.5, 23.6, 16.2; MS (ESI) $[M+H]^+$ 352.1.

Compound 4. It was prepared from 4-(6-choloropyridin-3-yl)benzaldehyde and 1-chloroacetyl-4-methylpiperazine, following general methods A and B, as a dihydrochloric acid salt (yellow solid). ^{1}H NMR (400 MHz, D_2O): δ 8.63 (s, 1H), 8.12 (d, *J* = 7.6 Hz, 1H), 7.70 (d, *J* = 7.2 Hz, 2H), 7.61 (d, *J* = 7.2 Hz, 2H), 7.38 (d, *J* = 7.6 Hz, 1H), 4.71-4.66 (m, 1H), 4.52-4.38 (m, 1H), 4.36-4.18 (m, 1H), 4.07-3.81 (m, 1H), 3.59-3.38 (m, 4H), 3.17-2.93 (m, 4H), 2.84 (s, 3H), 1.55-1.37 (m, 2H); ^{13}C NMR (100 MHz, DMSO-d^{6}): 165.2, 150.6, 143.3, 141.7, 135.2, 134.5, 130.6, 127.0, 126.8, 120.1, 52.8, 48.7, 48.9, 45.9, 36.0, 23.5, 16.5; MS (ESI) $[M+H]^+$ 385.2.

Compound 5. It was prepared from 2-bromobenzaldehyde and 1-chloroacetyl-4-methylpiperazine, following general methods A and B, as a dihydrochloric acid salt (white solid). ^{1}H NMR (400 MHz, DMSO-d6): δ 11.65-11.54 (bs, 1H), 9.79-9.70 (bs, 1H), 7.60 (d, *J* = 7.2 Hz, 1H), 7.32 (d, *J* = 7.2 Hz, 1H), 7.29-7.10 (m, 2H), 4.40-4.21 (m, 2H), 3.91-3.87 (m, 1H), 3.87-3.02 (m, 8H), 2.80 (s, 3H), 2.73-2.69 (m, 1H), 1.63-1.56 (m, 1H), 1.25-1.16 (m, 1H) ; ^{13}C NMR (100 MHz, DMSO-d^{6}): 164.4, 137.2, 132.8, 127.3, 127.2, 121.1, 51.6, 49.4, 48.3, 46.0, 36.7, 23.0, 16.4; MS (ESI) $[M+H]^+$ 352.1.

Compound 6. It was prepared from 3-bromobenzaldehyde and 1-chloroacetyl-4-methylpiperazine, following general methods A and B, as a dihydrochloric acid salt (white solid). ^{1}H NMR (400 MHz, DMSO-d6): δ 11.72-11.64 (bs, 1H), 9.84-9.76 (bs, 1H), 7.42- 7.38 (m, 2H), 7.22 (d, *J* = 8.0 Hz, 1H), 7.17 (d, *J* = 8.0 Hz, 1H), 4.39-4.32 (m, 2H), 4.20-3.98 (m, 1H), 3.68-3.05 (m, 8H), 2.73 (s, 3H), 2.70-2.59 (m, 1H), 1.62-1.58 (m, 1H), 1.30-1.26 (m, 1H); ^{13}C NMR (100 MHz, DMSO-d^{6}): 164.4, 141.8, 130.3, 128.6, 128.0, 124.1, 123.3, 51.4, 49.3, 48.7, 45.1, 36.2, 23.3, 16.0; MS (ESI) $[M+H]^+$ 352.1.

Compound 7. It was prepared from 4-benzyloxy-benzaldehyde, following the general methods A and C, as a hydrochloric acid salt (white powder). ^{1}H NMR (400 MHz, *d6*-DMSO): δ 8.32 (bs, 2H), 7.44-7.26 (m, 5H), 7.08 (d, *J* = 8.8 Hz, 2H), 6.93 (d, *J* = 8.8 Hz, 2H), 5.07 (s, 2H), 2.74-2.65 (m, 1H), 2.29-2.15 (m, 1H), 1.55-1.43 (m, 1H), 1.29-1.22 (m, 1H); ^{13}C NMR (100 MHz, DMSO-d^{6}): 154.3, 136.1, 133.2, 128.6, 128.0, 127.6, 126.4, 113.6, 71.4, 34.5, 25.5, 17.9; MS (ESI) $[M+H]^+$ 240.1.

Compound 8. It was prepared from 4-(6-fluoropyridin-3-yl)benzaldehyde, following the general methods A and C, as a dihydrochloric acid salt (grey solid). ^{1}H NMR (400 MHz, DMSO-d6): δ 8.29 (s, 1H), 8.10 (t, *J* = 8.0 Hz, 1H), 7.51 (d, *J* = 8.0 Hz, 2H), 7.18 (d, *J* = 8.0 Hz, 2H), 7.07 (d, *J* = 8.8 Hz, 1H), 2.82-2.75 (m, 1H), 2.37-2.30 (m, 1H), 1.38-1.30 (m, 1H), 1.29-1.22 (m, 1H); ^{13}C NMR (100 MHz, DMSO-d^6): 160.9, 141.2, 135.3, 133.6, 129.3, 127.1, 126.8, 111.3, 34.2, 25.8, 17.8; MS (ESI) $[M+H]^+$ 229.1.

Compound 9. It was prepared from 4-bromobenzaldehyde, following the general methods A and C, as a free amine (light yellow solid). ^{1}H NMR (400 MHz, $CDCl_3$): δ 7.35 (d, *J* = 8.4 Hz, 2H), 6.89 (d, *J* = 8.4 Hz, 2H), 2.53-2.48 (m, 1H), 1.90-1.84 (m, 1H), 1.09-1.04 (m, 1H), 0.96-0.93 (m, 1H); ^{13}C NMR (100 MHz, DMSO-d^6): 141.3, 130.1, 128.8, 117.8, 34.5, 25.3, 17.6; MS (ESI) $[M+H]^+$ 212.0.

Compound 10. It was prepared from 4-bromobenzaldehyde and 1-chloroacetyl-4-Boc-piperazine, following general methods A and B, as a dihydrochloric acid salt (yellow powder). ^{1}H NMR (400 MHz, DMSO-d6): δ 9.08-8.87 (m, 3H), 7.46 (d, *J* = 8.4 Hz, 2H), 7.12 (d, *J* = 8.4 Hz, 2H), 4.19 (s, 2H), 3.67-3.56 (m, 4H), 3.15-3.08 (m, 3H), 3.08-3.03 (m, 2H), 2.67-2.62 (m, 1H), 1.50-1.44 (m, 1H), 1.24-1.18 (m, 1H) ; ^{13}C NMR (100 MHz, DMSO-d^6): 164.4, 139.2, 130.6, 126.7, 119.9, 51.9, 48.9, 47.6, 36.3, 23.2, 16.5; MS (ESI) $[M+H]^+$ 338.1.

Compound 11. It was prepared from 4-bromobenzaldehyde and N-chloroacetylpiperidine, following general methods A and B, as a hydrochloric acid salt (yellow powder). ^{1}H NMR (400 MHz, DMSO-d6): δ 8.40-7.62 (bs, 2H), 7.45 (d, *J* = 8.0 Hz, 2H), 7.10 (d, *J* = 8.0 Hz, 2H), 4.11 (s, 2H), 2.79-2.73 (m, 1H), 2.59-2.54 (m, 1H), 2.26-2.20 (m, 1H), 1.89-1.82 (m, 1H), 1.34-1.00 (m, 8H) ; ^{13}C NMR (100 MHz, DMSO-d^6): 167.2, 139.3, 130.5, 126.7, 119.9, 48.9, 48.2, 36.8, 25.4, 24.8, 22.4, 14.7; MS (ESI) $[M+H]^+$ 337.1.

Compound 12. It was prepared from 4-bromobenzaldehyde and N-chloroacetyl pyrrolidine, following general methods A and B, as a hydrochloric acid salt (brown powder). ^{1}H NMR (400 MHz, DMSO-d6):

δ 9.20-8.85 (bs, 2H), 7.46 (d, *J* = 8.4 Hz, 2H), 7.11 (d, *J* = 8.4 Hz, 2H), 4.00 (s, 2H), 3.41-3.39 (m, 2H), 2.86-2.79 (m, 1H), 2.43-2.35 (m, 2H), 1.90-1.83 (m, 2H), 1.82-1.73 (m, 2H), 1.49-1.41 (m, 1H), 1.27-1.19 (m, 2H); ^{13}C NMR (100 MHz, DMSO-d^6): 167.6, 139.8, 130.8, 126.3, 119.2, 48.5, 48.1, 36.1, 25.2, 22.0, 14.4; MS (ESI) $[M+H]^+$ 323.1.

Compound 13. It was prepared from benzaldehyde and 1-chloroacetyl-4-Boc-piperazine, following general methods A and B, as a dihydrochloric acid salt (yellow powder). ^{1}H NMR (400 MHz, DMSO-d6): δ 9.61-9.35 (bs, 3H), 7.33-7.26 (m, 2H), 7.25-7.21 (m, 1H), 7.19-7.12 (m, 2H), 4.27 (s, 2H), 3.77-3.61 (m, 5H), 3.19-3.12 (m, 2H), 3.10-3.03 (m, 2H), 2.91-2.84 (m, 1H), 1.60-1.53 (m, 1H), 1.32-1.21 (m, 1H); ^{13}C NMR (100 MHz, DMSO-d^6): 164.4, 139.9, 128.3, 127.8, 125.4, 51.8, 48.7, 47.5, 36.7, 23.3, 16.6; MS (ESI) $[M+H]^+$ 260.2.

Compound 14. It was prepared from benzaldehyde and N-chloroacetylpiperidine, following general methods A and B, as a hydrochloric acid salt (yellow powder). ^{1}H NMR (400 MHz, DMSO-d6): δ 9.28-9.18 (bs, 1H), 7.33-7.27 (m, 2H), 7.25-7.21 (m, 1H), 7.19-7.15 (m, 2H), 4.20 (s, 2H), 3.70-3.66 (m, 1H), 3.48 (*dd*, *J* = 12.8 & 5.6 Hz, 2H), 3.19-3.12 (m, 1H), 3.10-3.03 (m, 1H), 2.91-2.84 (m, 1H), 1.75-1.20 (m, 8H) ; ^{13}C NMR (100 MHz, DMSO-d^6): 167.0, 140.1, 128.1, 127.3, 125.1, 50.6, 48.7, 36.2, 25.4, 24.6, 23.0, 15.2; MS (ESI) $[M+H]^+$ 259.2.

Compound 15. It was prepared from 4-bromobenzaldehyde and N-Boc-4-formylpiperidine, following general methods A and C, as a di-trifluoroacetic acid salt (yellow oil). ^{1}H NMR (400 MHz, $CDCl_3$): δ 7.34 (d, *J*= 8.4 Hz, 2H), 6.88 (d, *J*= 8.4 Hz, 2H), 2.59 (dd, *J* = 6.0 & 2.4 Hz, 2H), 2.30-2.25 (m, 1H), 1.84-1.78 (m, 1H), 1.74-1.55 (m, 4H), 1.36-1.22 (m, 2H), 1.18-1.01 (m, 4H), 0.96-0.89 (m, 1H); ^{13}C NMR (100 MHz, DMSO-d^6): 140.5, 131.7, 129.3, 120.1, 50.7, 46.2, 37.6, 36.6, 28.8, 23.3, 16.2; MS (ESI) $[M+H]^+$ 309.1.

Compound 16. It was prepared from benzaldehyde and N-Boc-4-formylpiperidine, following general methods A and C, as a di-trifluoroacetic acid salt (yellow oil). ^{1}H NMR (400 MHz, DMSO-d6): δ 8.95-8.22 (m, 4H), 7.36-7.02 (m, 5H), 3.38-3.11 (m, 2H), 3.04-2.62 (m, 4H), 2.35-2.21 (m, 1H), 2.03-1.10 (m, 8H); ^{13}C NMR (100 MHz, DMSO-d^{6}): 140.6, 128.1, 127.7, 125.1, 50.4, 46.5, 37.2, 36.5, 29.0, 23.8, 15.8; MS (ESI) [M+H]$^{+}$ 231.2.

Compound 17. It was prepared from 4-bromobenzaldehyde and Benzyl chloride, following general methods A and B, as a hydrochloric acid salt (white powder). ^{1}H NMR (400 MHz, DMSO-d6): δ 8.93 (bs, 2H), 7.58-7.21 (m, 7H), 7.05 (d, *J* = 8.0 Hz, 2H), 4.24 (s, 2H), 2.92-2.78 (m, 1H), 1.62-1.56 (m, 1H), 1.38-1.17 (m, 2H); ^{13}C NMR (100 MHz, DMSO-d^{6}): 140.7, 140.1, 131.3, 129.3, 128.6, 127.9, 120.2, 51.6, 36.9, 23.7, 16.1; MS (ESI) [M+H]$^{+}$ 302.1.

Compound 18. It was prepared from 4-bromobenzaldehyde and 4-hydroxybenzaldehyde, following general methods A and C, as a hydrochloric acid salt (white powder). ^{1}H NMR (400 MHz, DMSO-d6): δ 7.75 (d, *J* = 8.4 Hz, 2H), 7.37 (d, *J* = 8.0 Hz, 2H), 6.96 (d, *J* = 8.0 Hz, 2H) , 6.79 (d, *J* = 8.4 Hz, 2H) , 3.60 (s, 2H), 2.38-2.30 (m, 1H), 1.78-1.74 (m, 1H), 0.98-0.89 (m, 2H); ^{13}C NMR (100 MHz, DMSO-d^{6}): 156.5, 140.6, 140.1, 133.0, 131.7, 130.8, 129.4, 128.6, 120.1, 116.0, 51.7, 37.1, 23.6, 15.8; MS (ESI) [M+H]$^{+}$ 318.1.

Compound 19. It was prepared from 4-bromobenzaldehyde and 4-aminobenzaldehyde, following general methods A and C, as a dihydrochloric acid salt (white powder). ^{1}H NMR (400 MHz, DMSO-d6): δ 7.43-7.34 (m, 4H), 7.03 (d, *J* = 8.4 Hz, 2H), 6.94 (d, *J* = 8.4 Hz, 2H), 3.91(s, 2H), 2.63-2.57 (m, 1H), 1.85-1.60 (m, 1H), 0.89-0.84 (m, 2H); ^{13}C NMR (100 MHz, DMSO-d^{6}): 146.9, 140.4, 131.2, 129.4, 128.6, 128.2, 119.8, 114.8, 51.7, 37.3, 23.3, 15.8; MS (ESI) [M+H]$^{+}$ 317.1.

Compound 20. It was prepared from 4-bromobenzaldehyde and 4-(dimethylamino)benzaldehyde, following general methods A and C, as a dihydrochloric acid salt (white powder). ^{1}H NMR (400 MHz,

DMSO-d6): δ 7.46-7.36 (m, 3H), 7.09-7.05 (m, 2H), 7.00 (d, *J* = 8.4 Hz, 1H), 6.94 (d, *J* = 8.4 Hz, 1H) , 6.63 (d, *J* = 8.4 Hz, 1H) , 3.67 (s, 2H), 2.84 (s, 6H), 2.18-2.16 (m, 1H), 1.79-1.76 (m, 1H), 1.01-0.93 (m, 2H); ^{13}C NMR (100 MHz, DMSO-d^6): 148.2, 140.3, 131.2, 129.4, 128.4, 128.2, 119.6, 112.2, 51.9, 41.5, 37.1, 23.5, 15.2; MS (ESI) $[M+H]^+$ 345.1.

Compound 21. It was prepared from 4-bromobenzaldehyde and isonicotinaldehyde, following general methods A and C, as a dihydrochloric acid salt (white powder). ^1H NMR (400 MHz, DMSO-d6): δ 8.46 (d, *J* = 4.8 Hz, 2H), 7.40-7.30 (m, 4H), 7.01-6.98 (m, 1H), 6.92 (d, *J* = 8.4 Hz, 1H), 3.78 (s, 2H), 2.20-2.18 (m, 1H), 1.82-1.77 (m, 1H), 1.04-0.92 (m, 2H); ^{13}C NMR (100 MHz, DMSO-d^6): 150.3, 147.6, 140.1, 131.4, 129.3, 122.3, 119.5, 51.7, 37.3, 23.3, 15.1; MS (ESI) $[M+H]^+$ 303.1.

Compound 22. To a solution of compound **9** (freshly prepared, 42.2 mg, 0.2 mmol) in 2 mL of *anhydrous* CH_2Cl_2, K_2CO_3 (69.0 mg, 0.5 mmol) was added with stirring, after the addition, Acetic anhydride (20.6 mg, 0.2 mmol) in 1.0 mL of *anhydrous* CH_2Cl_2 was added with stirring, the reaction mixture was go on stirring for 1 h, quenched with water, extracted with CH_2Cl_2 (3 X 30 mL), dried over anhydrous Na_2SO_4, concentrated in vacuum to get a crude product which was further purified by column (EtOAc: *n*-hexane from 1:10 to 1:3) to get 48.2 mg (0.19 mmol, 95 % yield) of **compound 22** as a white solid. ^1H NMR (400 MHz, DMSO-d6): δ 7.38 (d, *J* = 8.0 Hz, 2H), 7.04 (d, *J* = 8.0 Hz, 2H), 5.70 (s, 1H), 2.85-2.80 (m, 1H), 2.04-1.98 (m, 1H), 1.98 (s, 3H), 1.31-1.22 (m, 2H); ^{13}C NMR (100 MHz, DMSO-d^6): 169.2, 141.1, 131.3, 129.35, 119.3, 33.1, 23.5, 22.6, 14.2; MS (ESI) $[M+H]^+$ 254.0.

Compound 23. To a solution of compound **9** (freshly prepared, 42.2 mg, 0.2 mmol) in 2 mL of *anhydrous* CH_2Cl_2, isocyanic acid benzyl ester (24.0 mg, 0.2 mmol) in 1.0 mL of *anhydrous* CH_2Cl_2 was added with stirring, the reaction mixture was go on stirring for 1 h, quenched with water, extracted with CH_2Cl_2 (3 X 30 mL), dried over anhydrous Na_2SO_4, concentrated in vacuum to get a crude product which was further purified by column (EtOAc: *n*-hexane from 1:10 to 1:3) to get 66.0 mg (0.20 mmol, 99 % yield) of **compound 23** as a brown solid. ^1H NMR (400 MHz, DMSO-d6): δ 7.68 (s, 1H), 7.43-7.26 (*m*, 6H), 7.12-

6.87 (m, 3H), 6.35 (s, 1H), 3.15-2.96 (m, 1H), 2.18-2.05 (m, 1H), 1.31-1.21 (m, 2H); ^{13}C NMR (100 MHz, DMSO-d^6): 152.5, 141.5, 140.1, 131.3, 129.4, 128.7, 128.0, 121.3, 119.2, 33.3, 23.1, 15.2; MS (ESI) $[M+H]^+$ 331.0.

Compound 24. It was prepared from 4-bromobenzaldehyde and benzaldehyde, following general methods A and C, as yellow oil. ^{1}H NMR (400 MHz, DMSO-d6): δ 8.41 (s, 1H), 7.74-7.62 (m, 2H), 7.42-7.36 (m, 5H), 7.00 (d, *J* = 8.0 Hz, 2H), 3.16-3.11 (m, 1H), 2.51-2.43 (m, 1H), 1.71-1.62 (m, 1H), 1.47-1.38 (m, 1H); ^{13}C NMR (100 MHz, DMSO-d^6): 161.6, 142.6, 136.7, 131.4, 131.1, 129.3, 128.4, 119.6, 35.7, 23.0, 14.8; MS (ESI) $[M+H]^+$ 300.1.

Compound 25. It was prepared from 4-bromobenzaldehyde, following general methods A and C, as a yellow oil. ^{1}H NMR (400 MHz, $CDCl_3$): δ 8.34 (s, 1H), 7.53 (d, *J*= 7.2 Hz, 2H), 7.38 (d, *J* = 7.6 Hz, 2H), 7.25 (d, *J*= 7.2 Hz, 2H), 6.97 (d, *J* = 7.6 Hz, 2H), 3.16-3.08 (m, 1H), 2.50-2.42 (m, 1H),1.69-1.62 (m, 1H), 1.48-1.39 (m, 1H); ^{13}C NMR (100 MHz, DMSO-d^6): 161.7, 142.3, 134.1, 131.2, 129.5, 128.4, 125.3, 119.7, 35.5, 23.2, 14.8; MS (ESI) $[M+H]^+$ 377.9.

Compound 26. It was prepared from 4-bromobenzaldehyde and 2-methoxylbenzaldehyde, following general methods A and C, as yellow oil. ^{1}H NMR (400 MHz, $CDCl_3$): δ 8.83 (s, 1H), 7.38 (d, *J*= 7.2 Hz, 2H), 7.36-7.28 (m, 2H), 7.21-7.18 (m, 1H), 6.98 (d, *J* = 7.2 Hz, 2H), 6.90 (d, *J* = 8.4 Hz, 1H), 3.86 (s, 3H), 3.22-3.16 (m, 1H), 2.52-2.42 (m, 1H),1.69-1.60 (m, 1H), 1.49-1.39 (m, 1H) ; ^{13}C NMR (100 MHz, DMSO-d^6): 164.2, 162.6, 141.9, 131.2, 130.7, 128.5, 128.1, 119.2, 114.1, 54.2, 35.5, 22.6, 14.3; MS (ESI) $[M+H]^+$ 330.0.

Compound 27. It was prepared from 4-bromobenzaldehyde and 3-methoxylbenzaldehyde, following general methods A and C, as yellow oil.^{1}H NMR (400 MHz, $CDCl_3$): δ 8.38 (s, 1H), 7.39 (d, *J*= 7.2 Hz, 2H), 7.34-7.20 (m, 3H), 7.08-6.89 (m, 3H), 3.88 (s, 3H), 3.23-3.18 (m, 1H), 2.50-2.41 (m, 1H), 1.48-1.40

(m, 2H); ^{13}C NMR (100 MHz, DMSO-d^6): 163.6, 160.8, 142.1, 140.2, 131.5, 130.2, 121.2, 119.2, 116.4, 112.0, 54.8, 35.8, 22.1, 14.2; MS (ESI) $[M+H]^+$ 330.0.

Compound 28. It was prepared from 4-bromobenzaldehyde and 3, 4, 5-trimethoxylbenzaldehyde, following general methods A and C, as yellow oil. ^{1}H NMR (400 MHz, $CDCl_3$): δ 8.29 (s, 1H), 7.38 (d, *J*= 8.0 Hz, 2H), 6.98 (d, *J*= 8.0 Hz, 2H), 6.93 (s, 2H), 3.94-3.85 (m, 9H), 3.12-3.08 (m, 1H), 2.49-2.41 (m, 1H), 1.64-1.60 (m, 1H), 1.42-1.33 (m, 1H); ^{13}C NMR (100 MHz, DMSO-d^6): 161.2, 158.2, 151.6, 142.9, 142.1, 131.4, 130.1, 125.1, 119.3, 116.7, 106.1, 61.1, 60.5, 58.2, 35.1, 20.9, 13.4; MS (ESI) $[M+H]^+$ 390.0.

Compound 29. It was prepared from 4-bromobenzaldehyde and 4-(dimethylamino)benzaldehyde, following general methods A and C, as a pale yellow oil. ^{1}H NMR (400 MHz, $CDCl_3$): δ 8.33 (s, 1H), 7.57 (d, *J*= 8.0Hz, 2H), 7.37 (d, *J*= 8.0 Hz, 2H), 6.97 (d, *J*= 8.4Hz, 2H), 6.67 (d, *J*= 8.4Hz, 2H), 3.00 (s, 6H), 2.48-2.36 (m, 1H), 1.63-1.51 (m, 1H), 1.36-1.11 (m, 2H); ^{13}C NMR (100 MHz, DMSO-d^6): 163.6, 163.6, 151.7, 140.9, 131.5, 129.7, 125.8, 124.6, 119.4, 111.4, 41.1, 34.7, 22.3, 14.1; MS (ESI) $[M+H]^+$ 343.1.

Compound 30. It was prepared from 4-bromobenzaldehyde and isonicotinaldehyde, following general methods A and C, as a pale yellow oil. ^{1}H NMR (400 MHz, $CDCl_3$): δ 8.74-8.60 (bs, 2H), 8.36 (s, 1H), 7.66-7.51 (bs, 2H), 7.39 (d, *J*= 8.4 Hz, 2H), 6.98 (d, *J*= 8.0Hz, 2H), 3.18-3.16 (m, 1H), 2.52-2.50 (m, 1H), 1.72-1.69 (m, 1H), 1.50-1.47 (m, 1H); ^{13}C NMR (100 MHz, DMSO-d^6): 163.3, 151.8, 149.1, 141.1, 131.2, 129.2, 120.4, 124.6, 119.5, 34.6, 22.5, 14.3; MS (ESI) $[M+H]^+$ 301.0.

Compound 31. To a solution of styrene (3.10 g, 30.0 mmol), powered KOH (2.50 g, 45.0 mmol), tetra-*n*-butylammonium bromide (300 mg) in CH_2Cl_2 (15 mL), bromoform (11.39 g, 45.0 mmol) was added dropwise at 40 °C in 1 h. The reaction mixture was stirred for 2 h at 25 °C, then filtered through a short silica gel column, concentrated under reduced pressure, and purified by column chromatography (silica gel, hexane) to give compound **41** (5.32 g, 90% yield). ^{1}H NMR (400 MHz, $CDCl_3$): δ 7.39-7.29 (m, 3H),

7.28-7.23 (m, 2H), 2.97 (dd, J = 10.0 & 8.0 Hz, 1H), 2.14 (dd, J = 10.0 & 8.0 Hz, 1H), 2.02 (t, J = 8.0 Hz, 1H).

EtMgBr (3 M in Et_2O, 5 mL, 15.0 mmol) was added dropwise to a solution of compound **41** (2.76 g, 10.0 mmol) and $Ti(Oi\text{-}Pr)_4$ (57.0 mg) in Et_2O (30 mL) at 25 ºC. The reaction mixture was stirred for 30 min, quenched with 10% H_2SO_4 (20 mL) and extracted with Et_2O (3 x 50 mL). The combined organic layers were washed with brine, dried over Na_2SO_4, filtered and concentrated. Column chromatography (silica gel, hexane) gave compound **42** as colorless oil (832 mg, 42% yield). ^1H NMR (400 MHz, $CDCl_3$): δ 7.37-7.18 (m, 5H), 3.35-3.23 (m, 1H), 2.36-2.24 (m, 1H), 1.62-1.51 (m, 1H), 1.36-1.26 (m, 1H).

The Grignard reagent from compound **42** (2 g, 10.0 mmol) and Mg (240.0 mg, 10.0 mmol) was prepared under a standard condition in anhydrous THF (20 mL), which was cooled to -78 ºC and a solution of di-*tert*-butyl azodicarboxylate (2.28 g, 10.0 mmol) in THF (5 mL) was added dropwise. The reaction mixture was stired for another 30 min, quenched with saturated NH_4Cl (20 mL), extracted with EtOAc (3 x 50 mL). The combined organic layers were washed with brine, dried over Na_2SO_4, filtered and concentrated. Column chromatography (silica gel, EtOAc: *n*-hexane from 1:10 to 1:3) gave compound **43** as a pale solid (2.44 g, 70% yield). ^1H NMR (400 MHz, $CDCl_3$): δ 7.25-7.22 (m, 3H), 7.20-7.09 (m, 2H), 6.42-5.95 (m, 1H), 3.56-3.30 (m, 1H), 3.19-3.05 (m, 0.35H), 2.33-2.20 (m, 0.65H), 1.52-1.42 (m, 19H), 1.27-1.22 (m, 1H).

Deprotection of compound **43**, following the procedure described in the general method B, afforded the hydrochloric acid salt of compound **31** as a white powder. ^1H NMR (400 MHz, DMSO-d6): δ 9.36 (bs, 1H), 8.72 (bs, 1H), 8.51 (bs, 1H), 7.35-7.22 (m, 3H), 7.20-7.11 (m, 2H), 3.55-3.28 (m, 1H), 2.45-2.10 (m, 1H), 1.50-1.41 (m, 1H), 1.29-1.20 (m, 1H); ^{13}C NMR (100 MHz, DMSO-d^6): 142.0, 128.2, 127.7, 125.1, 43.1, 20.6, 11.8; MS (ESI) $[M+H]^+$ 149.1.

Compound 32: It was prepared from compound **43** and 1-chloroacetyl-4-Bocpiperazine following general methods B, as a trihydrochloric acid salt. ^{1}H NMR (400 MHz, DMSO-d6): δ 10.61-9.62 (m, 3H), 7.40-7.22 (m, 3H), 7.20-7.10 (m, 2H), 4.17 (s, 2H), 3.70-3.51 (m, 4H), 3.21-2.96 (m, 4H), 2.89-2.82 (m, 1H), 2.29-2.17 (m, 1H), 1.32-1.10 (m, 2H); ^{13}C NMR (100 MHz, DMSO-d^6): 164.5, 142.2, 128.2, 127.8, 125.2, 54.7, 51.9, 47.6, 40.1, 20.6, 12.1; MS (ESI) $[M+H]^+$ 275.2.

Compound 33: It was prepared from compound **43** and 4-Bocpiperazine-1-carbonyl chloride following general methods B, as a dihydrochloric acid salt. ^{1}H NMR (400 MHz, DMSO-d6): δ 9.85 (bs, 1H), 9.65 (bs, 1H), 9.19 (bs, 2H), 7.28-7.19 (m, 3H), 7.18-7.06 (m, 2H), 3.90-3.59 (m, 4H), 3.33-3.27 (m, 2H), 3.17-3.09 (m, 1H), 3.05-2.96 (m, 1H), 2.81-2.62 (m, 1H), 2.18-2.07 (m, 1H), 1.21-1.11 (m, 1H), 1.11-1.02 (m, 1H) ; ^{13}C NMR (100 MHz, DMSO-d^6): 161.1, 141.9, 128.3, 127.8, 125.0, 54.4, 47.4, 37.2, 21.5, 12.0; MS (ESI) $[M+H]^+$ 261.2.

Compound 34. The synthesis of compound **34** started from compound **40** (R^1 = 4-Br). To a solution of **40** (624.0 mg, 2.0 mmol) and pyridine (0.40 mL) in dry acetonitrile (15.0 mL), nitrosonium tetrafluoroborate ($NOBF_4$, 289.0 mg, 2.6 mmol) was added at -30 °C. The reaction mixture was warmed to 0 °C and stirred for 2 h before quenched with water. The product was extracted with EtOAc (3 x 30 mL) and organic phases were washed with 1 N HCl, saturated $NaHCO_3$ and brine, dried over Na_2SO_4, and evaporated to give an N-nitroso-substituted compound, which can be used directly used in next step. It was dissolved in MeOH (10.0 mL) and acetic acid (4.0 mL) and activated Zn power (1.20 g, 17.5 mmol) was added slowly at 0 °C. The reduction was complete in 8 h and the reaction mixture was filtered, basified with saturated $NaHCO_3$. The product was extracted with EtOAc (3 x 30 mL) and the combined organic layers were dried over Na_2SO_4 and concentrated. Column chromatography (silica gel, CH_2Cl_2 to CH_2Cl_2:MeOH = 10:1) to give N-amino-substituted compound (120.5 mg, 18.5% yield).

^{1}H NMR (400 MHz, $CDCl_3$): 7.38 (d, J = 8.0 Hz, 2H), 6.99 (d, J = 8.0 Hz, 2H), 2.99-2.93 (m, 1H), 2.25-2.16 (m, 1H), 1.51-1.39 (m, 10H), 1.18-1.10 (m, 1H).

Reductive amination of the compound thus obtained (the general method C) followed by Boc-deprotection (the general method B) afforded the trihydrochloric acid salt of compound **34** (off-white powder). ^{1}H NMR (400 MHz, DMSO-d6): δ 10.2-9.60 (m, 2H), 8.52-8.23 (m, 2H), 7.49-7.20 (m, 3H), 6.95 (d, J = 8.0 Hz, 2H), 4.24-4.13 (m, 2H), 3.88-3.62 (m, 4H), 3.18-2.84 (m, 5H), 1.32-0.95 (m, 2H); ^{13}C NMR (100 MHz, DMSO-d^6): 141.2, 132.5, 129.1, 119.2, 53.2, 46.8, 38.4, 35.1, 31.2, 20.4, 12.6; MS (ESI) $[M+H]^+$ 324.1.

Compound 35. A mixture of propargylamine (1.10 g, 20.0 mmol) and Boc_2O (3.40 g, 20.0 mmol) in of CH_2Cl_2 (20 mL) was stirred at 25 ºC for 12 h. It was then washed with 1 N HCl and the organic layer was dried over Na_2SO_4, concentrated, and recrystallized in CH_2Cl_2/hexane to give Boc-protected propargylamine (2.81 g, 91% yield). A mixture of the compound thus obtained (1.55 g, 10.0 mmol) and iodobenzene (2.04 g, 10.0 mmol), CuI (96.0 mg, 0.5 mmol) and $Pd(PPh_3)_2Cl_2$ (200.0 mg, 0.5 mmol) in CH_2Cl_2 (20 mL) and diisopropylethylamine (4.01 g, 30.0 mmol) was stirred at 25 ºC for 16 h before quenched with water. The Sonogashira coupling product was extracted with CH_2Cl_2 (2 x 25 mL) and the organic layers were washed with brine, dried over Na_2SO_4, and concentrated. Column chromatography (silica gel, EtOAc: *n*-hexane from 1:20 to 1:5) gave Boc-protected 3-phenyl-propargylamine (1.75 g, 76% yield). ^{1}H NMR (400 MHz, $CDCl_3$): δ 7.44-7.39 (m, 2H), 7.33-7.27 (m, 3H), 4.76 (s, 1H), 4.16 (s, 2H), 1.47 (s, 9H). The compound thus obtained was alkylated with 1-chloroacetyl-4-methylpiperazine and deprotected following the general method B to produce the dihydrochloric acid salt of compound **35** (white powder). ^{1}H NMR (400 MHz, DMSO-d6): δ 11.56 (s, 1H), 9.75 (bs, 2H), 7.54-7.50 (m, 2H), 7.48-7.42 (m, 3H), 4.47-4.20 (m, 2H), 4.13 (s, 2H), 3.94-3.85 (m, 2H), 3.26-3.19 (m, 4H), 3.12-3.01 (m, 1H), 2.99-2.90 (m, 1H), 2.76 (s, 3H); ^{13}C NMR (100 MHz, DMSO-d^6): 164.5, 131.8, 128.3, 128.0, 126.5, 85.3, 82.9, 51.8, 50.2, 48.7, 45.2, 36.6; MS (ESI) $[M+H]^+$ 272.2.
