# Supplementary material for: Structure activity relationship and modeling studies of inhibitors of lysine specific demethylase 1
Source: PLoS One. 2017 Feb 3;12(2):e0170301. doi: 10.1371/journal.pone.0170301 (PMC5291514; doi:10.1371/journal.pone.0170301)
Supplement: S1 Table — (PDF) [file pone.0170301.s003.pdf]

Table S1. Structures and LSD1 inhibitory activities of compounds **1-35** with embedded structures.

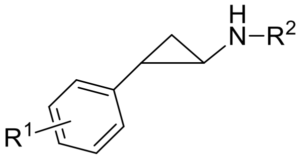

	R^1	R^2	LSD1 IC_{50} (μM)
**1**	4-OBn	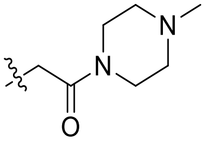	0.0098
**2**	4-(6-F-pyridin-3-yl)	same as above	0.077
**3**	4-Br	same as above	0.035
**4**	4-(6-Cl-pyridin-3-yl)	same as above	0.15
**5**	2-Br	same as above	>100
**6**	3-Br	same as above	>100
**7**	4-OBn	-H	0.91
**8**	6-F-pyridin-3-yl	-H	5.3
**9**	4-Br	-H	25
**10**	4-Br	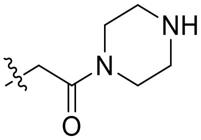	0.064
**11**	4-Br	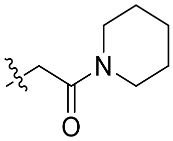	0.62
**12**	4-Br	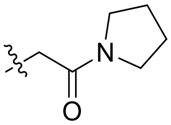	3.6
**13**	4-H	same as **10**	0.19
**14**	4-H	same as **11**	1.3
**15**	4-Br	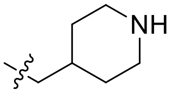	0.26
**16**	4-H	same as above	0.062
**17**	4-Br	-Bn	5.0

**18**	4-Br	-(4-OH)-Bn	94
**19**	4-Br	-(4-NH_2)-Bn	>100
**20**	4-Br	-(4-NMe_2)-Bn	71
**21**	4-Br	(pyridin-4-yl)-methyl	>100
**22**	4-Br	-$COCH_3$	15.4
**23**	4-Br	-CONHPh	7.4
**24**	4-Br	=CH-Ph	15.6
**25**	4-Br	=CH-(4-Br-Ph)	1.2
**26**	4-Br	=CH-(2-OMe-Ph)	0.74
**27**	4-Br	=CH-(3-OMe-Ph)	0.90
**28**	4-Br	=CH-(2,3,4-OMe-Ph)	94
**29**	4-Br	=CH-(4-NMe_2-Ph)	11
**30**	4-Br	=CH-(pyridine-4-yl)	14
**31**	4-H	-NH_2	5.8
**32**	4-H	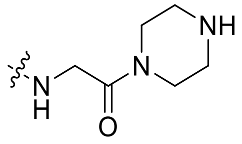	0.67
**33**	4-H	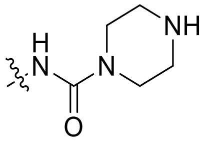	84
**34**	4-Br	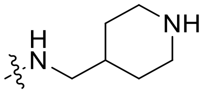	1.7
**35**	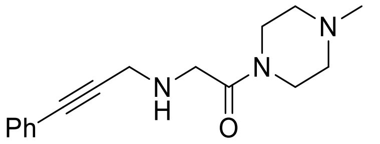		36.4
